# Is online objective structured clinical examination teaching an acceptable replacement in post-COVID-19 medical education in the United Kingdom?: a descriptive study

**DOI:** 10.3352/jeehp.2022.19.30

**Published:** 2022-11-07

**Authors:** Vashist Motkur, Aniket Bharadwaj, Nimalesh Yogarajah

**Affiliations:** 1 East and North Hertfordshire NHS Trust, Stevenage, UK; 2 University College London Medical School, London, UK; 3 Cambridge University Hospitals NHS Foundation Trust, Cambridge, UK; 4 West Hertfordshire Hospitals NHS Trust, Watford, UK; Hallym University, Korea

**Keywords:** Medical students, Distance education, Objective structured clinical examination, COVID-19, United Kingdom

## Abstract

**Purpose:**

Coronavirus disease 2019 (COVID-19) restrictions resulted in an increased emphasis on virtual communication in medical education. This study assessed the acceptability of virtual teaching in an online objective structured clinical examination (OSCE) series and its role in future education.

**Methods:**

Six surgical OSCE stations were designed, covering common surgical topics, with specific tasks testing data interpretation, clinical knowledge, and communication skills. These were delivered via Zoom to students who participated in student/patient/examiner role-play. Feedback was collected by asking students to compare online teaching with previous experiences of in-person teaching. Descriptive statistics were used for Likert response data, and thematic analysis for free-text items.

**Results:**

Sixty-two students provided feedback, with 81% of respondents finding online instructions preferable to paper equivalents. Furthermore, 65% and 68% found online teaching more efficient and accessible, respectively, than in-person teaching. Only 34% found communication with each other easier online; Forty percent preferred online OSCE teaching to in-person teaching. Students also expressed feedback in positive and negative free-text comments.

**Conclusion:**

The data suggested that generally students were unwilling for online teaching to completely replace in-person teaching. The success of online teaching was dependent on the clinical skill being addressed; some were less amenable to a virtual setting. However, online OSCE teaching could play a role alongside in-person teaching.

## Introduction

### Background

Restrictions due to the coronavirus disease 2019 (COVID-19) pandemic in 2020 had a significant impact on medical student teaching across universities [1]. Increased adoption of technology was needed to mitigate the risk of infecting students, vulnerable patients, and university staff, while ensuring that core competencies of medical school training were achieved [2-4].

Live and pre-recorded video tutorials were well-suited to be delivered over an online platform, with evidence showing them to be effective during the pandemic [5]. However, teaching and assessment in the style of objective structured clinical examinations (OSCEs) remain a central pillar of medical education. They present a greater logistical challenge, as they necessitate a high degree of interpersonal communication and practical skill demonstration to be able to target the “shows how” level of Miller’s pyramid [6]. They continue to be essential as a tool to mirror clinical scenarios both in formative settings to recognize poor performance, and in summative settings to assess the application of knowledge [7]. Although a wealth of information exists on traditional in-person OSCEs, the authors found scarce information on analyzing online OSCE teaching in medical education.

### Objectives

With the restrictions of the COVID-19 pandemic and the need to find an alternative to the classical OSCE setting, the authors developed a Surgical OSCE-Focused Teaching (“SOFT”) Series to trial the use of an online platform for teaching medical students in their first year of clinical studies at a UK medical school. We aimed to assess whether online teaching in the style of mock OSCE stations could ever replace in-person teaching and be accepted in the future training of undergraduate medical students.

## Methods

### Ethics statement

Ethical approval was not required for this study, as per the National Health Service Research Ethics Committee tool. This study did not include a clinical trial and did not collect any personal data. Completion of the feedback survey was optional for participants, and consent was received to use anonymous information to analyze the teaching series.

### Study design

The authors describe a descriptive study based on feedback via a post-teaching survey distributed to the students who had attended our teaching series. The findings are reported in the structure described by the STROBE (Strengthening the Reporting of Observational Studies in Epidemiology) checklist [8].

### Setting

This study was conducted at University College London (UCL) Surgical Society with students from UCL Medical School, between February and May 2021. Students were asked to complete an optional feedback survey (Supplement 1) via Google Forms (Google LLC, Mountain View, CA, USA) at the end of the teaching to assess the perceived characteristics of the session compared to their previous experiences of in-person teaching before COVID-19.

### Intervention

During the development of this OSCE series, the authors identified 6 common surgical topics that were likely to be seen in clinical practice (Table 1). Scenarios that would provide an adequate foundation upon which to test data interpretation, clinical knowledge, and communication skills were selected. The stations were independently verified by the Deputy Head of Year 4 MBBS (Bachelor of Medicine, Bachelor of Surgery) at the medical school for face validity and adjusted accordingly to be in keeping with the curriculum. Each clinical context was broken down into specific tasks, including history-taking, structured communication with healthcare professionals, imaging interpretation, safe prescription, low-fidelity assessment of an unwell patient, and developing management plans. The stations were piloted with a focus group, allowing the resolution of logistical issues and ambiguity in instructions. The final OSCE stations are available in Supplements 2–7.

The teaching series was delivered over Zoom (Zoom Video Communications Inc., San Jose, CA, USA), which included the use of breakout rooms to closely emulate the partitions present in an in-person OSCE. Each breakout room consisted of 3 to 4 students and 1 near-peer facilitator, who was an academic year above the students. The facilitator’s role was to keep time and guide the discussion and feedback. Read-only Google Docs (Google LLC) files containing instructions for each station were distributed to the participants.

Participants rotated through the role of student, patient, and examiner in the 6 stations and were given the opportunity to reflect on their experiences of each role. To simulate in-person OSCEs, participants were encouraged to keep their webcams on to maximize both verbal and visual interaction and facilitate a more natural conversation.

### Participants

Medical students in the first clinical year of their primary medical qualification at UCL Medical School were offered this teaching session via the student-led UCL Surgical Society and were invited to complete the post-teaching feedback survey. There were no exclusion criteria.

### Variables

The feedback survey requested participants’ opinions on the efficiency and accessibility of the format, the ease of understanding online instructions, the interaction with tutors and communication with peers through video calls, and whether the students would prefer this format to in-person teaching in the future. Participants were also asked in free-text fields for what they liked most about the series and for any suggestions for improvement.

### Data source/measurement

A 5-point Likert scale, from strongly disagree to strongly agree, was used for 6 questions as described above. These questions were assessed for face validity by the 3 authors. The internal reliability of the survey was calculated as Cronbach α=0.86. Responses were grouped together to give a combined percentage of “agree” and “strongly agree” responses. Comments from the 2 free-text fields were only edited for grammatical corrections by the two authors (V.M. and A.B.). The two authors (A.B. and V.M.) used open coding to generate initial codes for the free-text comments on the survey. The codes were reviewed to identify and define themes through further discussion between 2 authors (A.B. and V.M.). The themes defined for positive comments were “efficiency or convenience,” “interactivity,” and “general,” and those for negative comments were “difficulties with individual stations” and “general.” Examples of each of the themes highlighted by the participants were selected for inclusion in the results. Comments were not included if they were incomplete, were regarding specific tutors, or the content was covered by a comment already selected. The raw data can be found in Dataset 1.

### Bias

The teaching series was advertised via the social media channels of UCL Surgical Society and participants meeting the inclusion criteria were permitted to join. Completion of the feedback survey was optional for anyone who participated in the session as a student.

### Study size

There was no calculation of study size as any participant who met the inclusion criteria and had seen the announcement of the teaching session was permitted to join.

### Statistical methods

Descriptive statistics were used to analyze the Likert scale responses. Thematic analysis was used for the free-text responses.

## Results

### Participants

The feedback survey was sent to all 66 students who attended the online OSCE teaching series. A total of 62 responses were recorded over the course of the series, giving a response rate of 94.0%.

### Descriptive data

Descriptive data of the survey respondents were not collected; the responses were anonymous. All participants were in the first clinical year of their primary medical qualification at UCL Medical School.

### Main results

Forty respondents (64.5%) agreed or strongly agreed that the online OSCE format was more time-efficient than in-person seminar room teaching. Forty-two (67.7%) agreed or strongly agreed that this format was more accessible. Twenty-seven (43.6%) agreed or strongly agreed that interaction with tutors was easier through video calls and 21 (33.8%) agreed or strongly agreed that communication with peers was easier through video calls. Twenty-five (40.4%) agreed or strongly agreed that they would prefer this format of teaching over in-person seminar room teaching. Regarding online instructions, 50 (80.7%) of respondents found them easier to understand and retain than paper handouts (Table 2).

Positive free-text comments highlighted that the online format was efficient and convenient (Table 3). Some students found engagement through this medium to be positive. A few students valued the clarity of online resources. There were no comments suggesting that the online medium was better than in-person OSCE teaching, although some students did mention that the 2 methods could be used together.

Negative free-text comments expressed the unsuitability of some stations, such as prescribing and assessment of an unwell patient, in the online format (Table 4). Some respondents also highlighted that they would not want the online format in the future over in-person teaching.

## Discussion

### Key results

The format of online OSCEs used in this teaching session was found to be time efficient and accessible to the majority of survey respondents. The issues highlighted by the feedback were surrounding interaction with tutors and communication with colleagues. Twenty-five (40.4%) of respondents agreed that they would prefer the online format over in-person teaching. Free-text comments highlighted that the communication issues were more pertinent with certain stations that may be less suited to the online format.

### Interpretation

The clear benefit of running an OSCE teaching series online during the pandemic is infection control. Participants could access the session from their own homes and the development of the series was conducted remotely. The risk of COVID-19 to participants was minimized, as in other examples of online teaching [2-4]. No patients or professional actors were utilized in the teaching series, further minimizing exposure and protecting vulnerable individuals.

There was a predominant theme of efficiency and accessibility across the series. This may be due to the reduced cost and time of travel to the sessions. Similarly, an online OSCE using Zoom at Harvard School of Dental Medicine highlighted that students also valued the convenience of the online setup [9].

The authors found the main issue in the development of the stations to be the logistical limitations in teaching certain types of clinical scenarios. Despite the prior use of a focus group to uncover potential issues, our feedback revealed that students still found the assessment of an acutely unwell patient to be difficult over an online platform. The focus group also highlighted issues with a discarded joint examination station, in which they expressed that having to describe examination techniques felt cumbersome. Using such low-fidelity simulation settings, the suspension of disbelief is more difficult to attain online due to the lack of tactile and non-verbal feedback from other participants [1]. In our patient briefs, observation and examination findings were provided for this station; however, without a mannequin to examine, students found it difficult to elicit these findings. In this format they were only able to show attainment of the “knows how” level of Miller’s pyramid, rather than the “shows how” level [6].

The feedback the students received may have been limited as it omitted discussion of non-verbal cues when interacting online. Felthun et al. [10] reported that in this format, examiners would be unable to comment on positive and negative body language, which would otherwise play an important role in a real consultation. This inability to replicate some clinical scenarios could be detrimental to students’ learning as it does not address competencies that are essential for students. However, comments such as “the personalized feedback and small group setting was really useful” suggests that some students found online teaching less intimidating and were still able to learn and reflect from the process.

Through the absence of critical comments for some stations, it can be argued that certain clinical scenarios are well replicated in an online platform. History-taking is an example of a clinical competency that can be well assessed and reviewed in this format. Despite limited non-verbal communication, students still valued feedback on their questions and wording. Similarly, communication of management plans and interpretation of radiology results are other examples that can be well replicated online. The authors suggest that viewing radiology results on a screen is more akin to current clinical practice than reviewing print-outs. Kakadia et al. [9] reported that half of the respondents believed future OSCEs would be conducted online. As many aspects of medicine and medical education continue to transition in this way, teaching formats such as ours provide one more opportunity for students to further their online decorum.

Extrapolating from the student feedback, the authors suggest that there are other potential topics that could be explored in this online format. Data interpretation is one such topic. This could involve interpretation and explanation of blood test results, microbiology samples, arterial blood gas values or radiographs. Similarly, this modality could be extended to the assessment and description of fundoscopy, otoscopy, or skin lesions via the use of photographs. Other stations that focus on communication skills could also be incorporated. Conversations regarding the interface between ethics, law, and medicine relevant to the country in which the students are training are potential sources of stations. This could include contraception counseling, abortion, and capacity or end-of-life discussions.

Our results show that online platforms pose some obstacles in communication, which will be important to be aware of when developing stations for this format. They are likely a combination of non-verbal and verbal issues. Reasons could include connection difficulties, audio-visual lag, overlapping of voices, and difficulty replicating eye contact. Students in a previous study agreed that telehealth encounters, which could be comparable to the consultations in our OSCE stations, came with issues in maintaining eye contact and tone of voice [11]. However, our results also show that online handouts may be efficient in disseminating information, suggesting that despite barriers to online communication, it is possible to provide clear instructions through parallel means such as Google Doc links.

### Limitations

The authors acknowledge that the interpretation of the results from this paper is limited because the survey used to measure acceptance of the online format was assessed for face validity but not content validity. Since the students were not asked to directly compare an in-person teaching session with this online session, the responses of the students may be affected by recall bias. The use of an extensively validated survey could have given more objective data. This paper largely draws on the subjective comments of the participants.

This paper only collected data from a single medical school in London, England and as such, extrapolating to other medical settings across the globe may be limited due to factors such as access and exposure to technology and the internet. Further research involving other similar implementations of online OSCEs at other institutions will provide broader insights into student perceptions. This could be further expanded to studies where the same clinical stations are trialed in both online and in-person formats, which would then allow a direct comparison of the strengths and weaknesses of each. Additional research could be conducted incorporating the views of tutors as other stakeholders, which was not covered by this study.

As technology use in medical education further accelerates, virtual and mixed reality products could overcome some current limitations of online teaching. For example, a 3-dimensional augmented environment in which to visualize clinical signs, interpret body language, and simulate the use of clinical equipment would allow a wider range of stations than can be provided by an online session such as ours [12].

### Conclusion

This study tested a novel online OSCE approach to teach medical students in response to restrictions imposed by the COVID-19 pandemic. The teaching series was generally well received, with students commending the accessibility and efficiency of the sessions. Some clinical scenarios were well accepted in an online format. Others, such as examination and assessment of an unwell patient, were not. Overall, the data revealed that students were largely unwilling to completely replace in-person teaching with an online method. There is potential for this format to be accepted in conjunction with another in-person teaching. It would be interesting to further investigate how these views may change as online medical teaching becomes more prevalent and to expand the sample size with the addition of a multi-center analysis.

## Figures and Tables

**Table 1.** Description of the content of the objective structured clinical examination stations

Station no.	Clinical context	Task 1	Task 2
1	Dysphagia	Focused history-taking (5 min)	Communication with medical professionals using the “situation, background, assessment, recommendation” framework (3 min)
2	Fractured neck of femur	Interpreting and explaining a radiograph to a nursing student, including initial and definitive management (5 min)	Safe prescription of regular medications and analgesia using the World Health Organization Analgesic Ladder (5 min)
3	Scaphoid fracture	Interpreting and explaining a radiograph to a patient, including answering questions on further management (5 min)	–
4	Stomas	Matching clinical vignettes of conditions requiring ileostomy or colostomy and describing the differentiating features of each (3 min)	Analyzing a fluid balance chart and safe prescription of replacement and maintenance fluids (5 min)
5	Septic arthritis	Focused history-taking and conveying an initial management plan (5 min)	–
6	Postoperative sepsis	Assessment of an acutely ill patient using the “airway, breathing, circulation, disability, exposure” approach (10 min)	–

**Table 2.** Likert scale responses from the feedback survey from medical students at the University College London Medical School in the United Kingdom on the acceptability of the online objective structured clinical examination (n=62)

Item	Likert scale responses^a)^				
	1	2	3	4	5
I found this format of teaching more time efficient than in-person seminar room teaching	1 (1.6)	11 (17.7)	10 (16.1)	21 (33.9)	19 (30.6)
I found this format of teaching more accessible than previous in-person seminar room teaching	4 (6.5)	6 (9.7)	10 (16.1)	18 (29.0)	24 (38.7)
The online instructions and learning objectives were easier to understand and retain than paper handouts	1 (1.6)	4 (6.5)	7 (11.3)	22 (35.5)	28 (45.2)
I found it easier to interact with the tutors through video call than in-person	8 (12.9)	10 (16.1)	17 (27.4)	12 (19.4)	15 (24.2)
I found it easier to communicate within a group of peers through video call than in-person	9 (14.5)	13 (21.0)	19 (30.6)	10 (16.1)	11 (17.7)
I would prefer this format of teaching in the future rather than in-person seminar room teaching	5 (8.1)	15 (24.2)	17 (27.4)	13 (21.0)	12 (19.4)

Values are presented as number (%).

a) 1=strongly disagree, 5=strongly agree.

**Table 3.** Key contents of positive free-text comments

Topic of comment	Examples of positive comments
Efficiency or convenience	- Quick and efficient, well timed, mark schemes laid out well.
	- The personalized feedback and small group setting was really useful. It was convenient that it was on Zoom so there was no need to travel to a tutorial room.
	- Online is a big convenience—especially for OSCE teaching that doesn’t require practical things.
	- Could do it from my room and make notes. Really small groups, which I really appreciated, meant I could learn more.
Interactivity	- Love the interactivity! Admittedly I don’t really like situations like this but it has really been a lot of fun and very educational.
	- Smooth communication
General	- The variety of stations and the feedback was really helpful, some very good teaching points too.
	- Very clear mark-scheme
	- Google Docs was quick and easy.
	​​- Next best thing to face-to-face mock OSCE practice
	- I think this is an excellent addition to in-person practice opportunities.

OSCE, objective structured clinical examination.

**Table 4.** Key contents of negative free-text comments

Topic of comment	Examples of negative comments
Difficulties with individual stations	- Some of the stations didn’t work well in Zoom format, e.g., prescribing.
	- A-E assessments are quite hard to do online.
	- A-E assessments on zoom require a lot of imagination and acting skills, I think this is the only part I could find more helpful in person.
General	- I think in-person sessions in the future would be great if possible as we’d actually be able to practice examinations/have less technical issues.
	- Please don’t make video calls the standard method of teaching.
	- Maybe we fill in drug charts electronically then share an image/screen share our answer.

## Footnotes

**Data availability**
Data files are available from Harvard Dataverse: https://doi.org/10.7910/DVN/N3BLTZ Dataset 1. Raw response data from participants.

## Supplementary materials

Supplementary files are available from Harvard Dataverse: https://doi.org/10.7910/DVN/N3BLTZ

Supplement 1. Survey questionnaire used for the students to compare online teaching with previous experiences of in-person teaching.

Supplement 2. Surgical OSCE-Focused Teaching Station 1: 10 minutes—History and SBAR for 10 minutes, executed by the University College London (UCL) Surgical Society with students from UCL Medical School, between February and May 2021. SBAR, situation, background, assessment, recommendation.

Supplement 3. Surgical OSCE-Focused Teaching Station 2: 10 minutes—Radiology and Pain for 10 minutes, executed by the University College London (UCL) Surgical Society with students from UCL Medical School, between February and May 2021.

Supplement 4. Surgical OSCE-Focused Teaching Station 3: 5 minutes—Wrist Fractures for 5 minutes, executed by the University College London (UCL) Surgical Society with students from UCL Medical School, between February and May 2021.

Supplement 5. Surgical OSCE-Focused Teaching Station 4: 10 minutes—Stomas and Fluids for 10 minutes, executed by the University College London (UCL) Surgical Society with students from UCL Medical School, between February and May 2021.

Supplement 6. Surgical OSCE-Focused Teaching Station 5: 5 minutes—Red Hot Joint for 5 minutes, executed by the University College London (UCL) Surgical Society with students from UCL Medical School, between February and May 2021.

Supplement 7. Surgical OSCE-Focused Teaching Station 6: 10 minutes—DR ABCDE for 10 minutes, executed by the University College London (UCL) Surgical Society with students from UCL Medical School, between February and May 2021.

Supplement 8. Audio recording of the abstract.
