# Supplementary material for: Is online objective structured clinical examination teaching an acceptable replacement in post-COVID-19 medical education in the United Kingdom?: a descriptive study
Source: J Educ Eval Health Prof. 2022 Nov 7;19:30. doi: 10.3352/jeehp.2022.19.30 (PMC9807458; doi:10.3352/jeehp.2022.19.30)
Supplement: Supplementary file 2 — Supplement 1. Survey questionnaire used for the students to compare online teaching with previous experiences of in-person teaching. [file jeehp-19-30-suppl1.pdf]

Paperless SOFT 2021 Feedback

Thank you for attending our teaching session! We hope it has been a valuable use of your time and would be grateful if you could fill in this short feedback form in return, so that we can make improvements.

* Required

1. Name of tutor(s) *

____

2. I found this format of teaching more time efficient than in-person seminar room teaching

Mark only one oval.

	1	2	3	4	5	
Strongly Disagree	◯	◯	◯	◯	◯	Strongly Agree

3. I found this format of teaching more accessible than previous in-person seminar room teaching

Mark only one oval.

	1	2	3	4	5	
Strongly Disagree	◯	◯	◯	◯	◯	Strongly Agree

4. The online instructions and learning objectives were easier to understand and retain than paper handouts

Mark only one oval.

	1	2	3	4	5	
Strongly Disagree	◯	◯	◯	◯	◯	Strongly Agree

5. I found it easier to interact with the tutors through video call than in-person *

Mark only one oval.

	1	2	3	4	5	
Strongly Disagree	◯	◯	◯	◯	◯	Strongly Agree

6. I found it easier to communicate within a group of peers through video call than in-perso

Mark only one oval.

	1	2	3	4	5	
Strongly Disagree	◯	◯	◯	◯	◯	Strongly Agree

7. I would prefer this format of teaching in the future rather than in-person seminar room teaching

Mark only one oval.

	1	2	3	4	5	
Strongly Disagree	◯	◯	◯	◯	◯	Strongly Agree

8. What did you like the most about this teaching session? *

9. Do you have any suggestions for improvement? *

This content is neither created nor endorsed by Google.

Google Forms
