# Supplementary material for: Is online objective structured clinical examination teaching an acceptable replacement in post-COVID-19 medical education in the United Kingdom?: a descriptive study
Source: J Educ Eval Health Prof. 2022 Nov 7;19:30. doi: 10.3352/jeehp.2022.19.30 (PMC9807458; doi:10.3352/jeehp.2022.19.30)
Supplement: Supplementary file 3 — Supplement 2. Surgical OSCE-Focused Teaching Station 1: 10 minutes—History and SBAR for 10 minutes, executed by the University College London (UCL) Surgical Society with students from UCL Medical School, between February and May 2021. SBAR, situation, background, assessment, recommendation. [file jeehp-19-30-suppl2.pdf]

Station 1: 10 minutes

History and SBAR

Learning Objectives:

- Taking a history from a patient presenting with dysphagia
- Use the SBAR framework to effectively handover patient details

Task 1: History taking (5 mins)

- Spend 5 minutes taking a history from Mr Paul Stevens
  - There are information sheets for the student, patient and examiner
    - Student 1: Medical Student
    - Student 2: Patient
    - Student 3: Examiner

Feedback: 2 mins

Task 2: Communicating with medical professionals (3 mins)

- After taking the history, you decide to refer the patient to the **local hospital.**
- On clinical examination the patient is unwell and dehydrated.
- Another student should now **talk through** using the SBAR framework to the on call Surgical SpR to refer the patient.

Task 1: History taking

Student Brief

You are a medical student at a General Practice. Mr Paul Stevens, a 68 year old man comes in complaining of swallowing difficulties.

Please spend **5 minutes** taking a history from this patient (you do not need to do a review of systems).

Patient Brief

Presenting Complaint	Over the Christmas holidays I noticed that I was having trouble swallowing
Ideas, Concerns and Expectations	I'm worried I might have some kind of cancer.
History of Presenting Complaint	Provide this information if prompted to:<br>• *Onset: 3 weeks ago*<br>• *'stuck in the throat' not on initiation*<br>• *Solids/liquids: Initially solid foods but after water as well. It's now come to a point I can't eat anything.*<br>• *Gurgling: No*<br>• *Stable or worsening: It's got to the point where Ive hardly eaten anything in the past 4 days*<br>• *Pain: Uncomfortable feeling, no pain.*<br>• *Intermittent or continuous - continuous*<br>• *Level: above the stomach*<br>• *Alleviating: nothing has helped*<br>• *Trauma/foregin body: No trauma*<br>• *Halitosis: No bad breath*<br>• *Recent illnesses: N/A*<br>• *Previous episodes: N/A*<br>• *Unwell contacts: Nope*<br>• *Red flags:*<br>○ *Weight loss - yes 2kg in the past 3 weeks*<br>○ *Fever: No*<br>○ *Night sweats: No*
Past Medical History	Depression diagnosed 2 years ago<br>Had a fracture of the arm as he fell from a ladder at work
Drug History	Dx: Sertraline (depression diagnosed 2Y ago)<br><br>Allergies: Peanuts
Family History	Any upper GI cancer: No
Social History	Alcohol: drink only on weekends up to 8 pints of beer<br>Smoking: 10 cigarettes/day since the age of 20<br>Occupation: Work as a construction site manager
Review of Systems	None

Examiner brief

After the student has finished this task, please give feedback using the points below and outline anything the student may have missed.

Fail: When a student does not meet majority of the points in the borderline marking column

	Borderline	Additional points for Clear Pass
Introduction	Appropriate introduction and obtaining consent<br>Confirming patient details such as name and age	
Presenting Complaint	Encourages the patient to provide information using a few open questions	Starts with an open question.
Ideas, Concerns and Expectations		Explores ICE
History of Presenting Complaint	**Explores** some of the points covered below<br>● *Onset:* 3 weeks ago<br>● *Character:* Initially solid foods but after water as well. It's now come to a point I can't eat anything.<br>● *Time:* Has gotten worse since<br>● *Level:* above the stomach<br>● Alleviating: nothing has helped<br>● *Pain:* Uncomfortable feeling	**Ensures** that these important points are covered<br>● *Onset:* 3 weeks ago<br>● *Character:* Initially solid foods but after water as well. It's now come to a point I can't eat anything.<br>● *Time:* Has gotten worse since<br>● *Level:* above the stomach<br>● *Alleviating:* nothing has helped<br>● *Pain:* Uncomfortable feeling<br>● *Red flags:*<br>○ *Weight loss:* yes 2kg in the past 3 weeks<br>○ *Fever:* No<br>○ *Night sweats:* No<br><br>Asks about some of these less obvious symptoms<br>● *Trauma/foreign body:* No trauma<br>● *Halitosis:* No bad breath<br>● *Recent illnesses:* N/A<br>● *Previous episodes:* N/A<br>● *Unwell contacts:* No

		• *Gurgling*: No
Past Medical History	Ensures that the student asks the patient's about their PMH	
Drug History	Asks about their DHx<br>Enquires about patient's allergies	
Family History	Explores the patient's family history	Ensure that student enquiries about **relevant** FHx
Social History	Student asks about the following points<br>• Alcohol<br>• Smoking	Also asks:<br>• Occupation
Overall communication	Shows empathy<br>Maintains good eye contact and appropriate body language	Avoids technical jargon

Task 2: Communicating with medical professionals

Student Brief

- After taking the history, you decide to refer the patient to the local hospital.
- On clinical examination the patient is unwell and dehydrated.
- Choose one student to spend 3 minutes handing over to the on-call Surgical SHO (another student enacts this), using the SBAR framework.

Examination findings:
On examination of the neck, there was no swelling or pain.
You performed a fluid status exam and found dry mucous membranes.
HR: 110, regular
BP: 108/75
RR: 21

SBAR Framework
- Situation - who are you and why are you calling
- Background - summary of PC and HPC
- Assessment - your examination of the patient
- Recommendation - what you would like them to do for the patient, and what would they like you to do for the patient

Examiner Brief

Supervisor: After the student has finished this task, please give feedback using the points below and outline anything the student may have missed.

Summary:

Fail: *When a student does not meet majority of the points in the borderline marking column*

Borderline: *The student has used the SBAR framework reasonably well but could have been more structured and concise in their handover.*

Clear Pass: *The student has handed over the patient in a concise and structured manner with the relevant details.*

Situation:

Borderline	Clear Pass
Introduces themselves	Introduces themselves and makes sure they speaking to the on-call surgical team
Mentions patient demographics and presentation	Gives a concise opening summary about the patient and why they are contacting the surgeon

Model: Hi my name is ________ , from the local General Practice. Can I confirm that I am speaking to the on-call surgical SHO? I have a patient who is a 38 year old gentleman who is currently dehydrated and weak, secondary to dysphagia.
Could you please review the patient?

Background:

Borderline	Clear Pass
Reasonable summary of HPC	Concise and relevant summary of HPC with red flags symptoms highlighted

Model: I have a patient who has presented with a 3 week history of continuous dysphagia, on both solid and liquid foods, which has worsened over the weeks. This sensation of food getting stuck is accompanied by an uncomfortable sensation, just above the level of the stomach. There has also been a weight loss of 2kg and other than this, no relevant symptoms.

Assessment:

Borderline	Clear Pass
Presents examination and observation findings	Examination and observation findings presented in a fluid manner

On assessment, a fluid status examination revealed dry mucous membranes. The patient is tachycardic, tachypnea and borderline normal blood pressure. They are also not drowsy.

Recommendation:

Borderline	Clear Pass
Kindly requests a surgical review given the above findings	Candidate expressed concerns, requests a review and asks whether there is anything else they can do in the meantime

Model: I am worried that this patient may be experiencing dehydration due to their lack of oral intake. I would be grateful if you could review this patient with the on call surgical team in order to decide the best clinical course of action. Would you like me to do anything else in the meantime?
