# Supplementary material for: Is online objective structured clinical examination teaching an acceptable replacement in post-COVID-19 medical education in the United Kingdom?: a descriptive study
Source: J Educ Eval Health Prof. 2022 Nov 7;19:30. doi: 10.3352/jeehp.2022.19.30 (PMC9807458; doi:10.3352/jeehp.2022.19.30)
Supplement: Supplementary file 4 — Supplement 3. Surgical OSCE-Focused Teaching Station 2: 10 minutes—Radiology and Pain for 10 minutes, executed by the University College London (UCL) Surgical Society with students from UCL Medical School, between February and May 2021. [file jeehp-19-30-suppl3.pdf]

Station 2: 10 minutes

Radiology and Pain

Learning Objectives:

- Interpret a hip x-ray
- Describe the risks associated with a hip fracture
- Implement and understand the use of the analgesic ladder

Task 1: Patient who presents after a fall

- Spend 5 minutes on this task
    - One student to interpret and explain the x-ray findings to the nurse
    - One student acts as the nurse
    - One student is the examiner

Task 2: Patient in pain

- Spend 5 minutes prescribing appropriate medication for a patient in severe pain
    - One student explains their reasoning for what they would have prescribed, including dosage and timing, and which sections they would complete
    - Other two students to examine and give feedback based on the given mark scheme

Created by: Aniket Bharadwaj, Vashist Motkur and Nimalesh Yogarajah

Task 1: Patient who presents after a fall

Student Brief

You are a 4th year student in the emergency department. Mrs Jones, a 78 year old woman presents after a fall while walking her dog in her neighbourhood. She has a PMH of hypertension and Glaucoma. She takes Ramipril 10mg once daily and Timolol eye drops, one drop twice daily. She is allergic to codeine and clarithromycin.
She doesn't smoke or drink alcohol. Her most recent weight was 64kg.

One of the nursing students asks you to explain to her the x-ray below, please answer any other questions she may have.

Pelvic x-ray image redacted as permission not obtained for publication

Nurse brief

1. What does this x-ray show?
    a. *If a student fails to describe the fracture* - **How do you know her hip is broken?**
2. After the patient comes to A&E what steps would you be taking to manage them?
    a. If not mentioned ask: What do you do if the patient was in a lot of pain?
    b. Will she need surgery? If so, what would the procedure be?
3. Are there risks of surgery to the patient?

Examiner Brief

Fail: When a student does not meet majority of the points in the borderline marking column

Borderline	Clear Pass
Student identifies and describes as a fracture on the right hip<br><br>Names some of the major anatomical landmarks<br><br>Describes it as a displaced fracture	+ Explains by comparison to the other side<br><br>+ Names major landmarks - femur, head of femur, hip bone and R and L<br><br>+ Disruption of Shenton's line<br>+ Shortening of the bone
Will manage pain with medication (paracetamol or further up the pain ladder)<br><br>Senior review<br><br>Suggests definitive treatment would be surgery	+ Uses pain ladder and recognises opioids (e.g. oramorph) may be needed PRN alongside antiemetics PRN<br><br>+ Candidate explains that a neck of femur fracture must be managed surgically and that hip arthroplasty is the mainstay of treatment depending on patient morbidity<br>+ Excellent students may recognise it as a Garden Type 4 classification (complete fracture with complete

	displacement)
Common risks of surgery:<br>- Infection<br>- Bleeding	+ Post operative pain<br>+ Damage to surrounding anatomical structures<br>+ Scar formation<br>+ Failure of successful surgery, requiring further revision surgery<br><br>Excellent students may mention<br>+ VTE due to immobilisation<br>+ Lung collapse common after anaesthetic procedures

Task 2: Patient in pain

Student Brief

You are on the ward in the evening with the surgical team who have arranged for Mrs Jones to be operated on early tomorrow.

A nurse informs your team that the patient is in terrible pain.

Please explain to your team what you would like to prescribe for this patient.

She takes Ramipril 10mg once daily and Timolol eye drops, one drop twice daily.
Her most recent weight was 64kg. She is allergic to codeine and clarithromycin.

BNF can be used via this link: https://bnf.nice.org.uk/

Mock hospital drug chart image redacted as permission not obtained for publication

Created by: Aniket Bharadwaj, Vashist Motkur and Nimalesh Yogarajah

Examiner Brief

Fail:

- Incorrect patient data entered
- Only paracetamol considered for pain management
- Codeine prescribed despite allergy

Borderline Pass	Clear Pass
All patient details filled out	+ Regular medication prescribed<br>+ some students may exclude ramipril since the patient is 1 day pre op
Allergies noted	
Paracetamol prescribed correct dose	
NSAID correct dose (Ibuprofen 400mg TDS)	+ PPI cover (Omeprazole or Lanzoprazole)
Morphine (Oramorph 2.5mg-5mg 2-4hrly)	+ Laxative (Senna, Movicol or docusate sodium)<br>+ Antiemetic prescribed<br>Cyclizine 50mg TDS SC/IV/PO<br>*or*<br>Ondansetron 8mg TDS PO/IV<br>*or*<br>Metoclopramide 10mg<br><br>+ Some students may consider Tramadol 50mg-100mg QDS

Created by: Aniket Bharadwaj, Vashist Motkur and Nimalesh Yogarajah
