# Supplementary material for: Is online objective structured clinical examination teaching an acceptable replacement in post-COVID-19 medical education in the United Kingdom?: a descriptive study
Source: J Educ Eval Health Prof. 2022 Nov 7;19:30. doi: 10.3352/jeehp.2022.19.30 (PMC9807458; doi:10.3352/jeehp.2022.19.30)
Supplement: Supplementary file 5 — Supplement 4. Surgical OSCE-Focused Teaching Station 3: 5 minutes—Wrist Fractures for 5 minutes, executed by the University College London (UCL) Surgical Society with students from UCL Medical School, between February and May 2021. [file jeehp-19-30-suppl4.pdf]

Station 3: 5 minutes

Wrist Fractures

Learning Objectives:

- Explain management of a scaphoid fracture to a patient
    - *Be aware that in UCL Year 4 OSCEs, would will only be asked to act as a Year 4 Student - this station is asking you to act as a Junior Doctor*

Task 1: Patient discussion

- Spend 5 minutes talking to the patient about his radiograph findings and answering his questions

Created by: Aniket Bharadwaj, Vashist Motkur and Nimalesh Yogarajah

Student Brief

Mr Henry James is a 35 year old man who sustained an injury to his wrist at work. He was seen in the fracture clinic and had a wrist splint applied 2 weeks ago. Initial x-ray showed no abnormality. He was asked to come back to the fracture clinic for a repeat x-ray.

Please discuss the radiograph findings with the patient, and answer any questions he may have.

AP and lateral wrist radiographs redacted as permission not obtained for publication

Patient Brief

You are a 35 year old man who works on a construction site. You had a fall at work and injured your wrist 2 weeks ago. When you attended the fracture clinic they informed you that there was no fracture on the x-ray, but gave you a splint to wear for 2 weeks. You have attended your appointment today for a repeat x-ray.

You have the following concerns:

1. Why do I still have pain in my wrist?
2. (If the student states there is a fracture) Why was I previously incorrectly told there was no fracture?
3. What happens now? Will I need surgery?
4. How soon can I remove the cast/splint?

Examiner Brief

Fail: When a student does not meet majority of the points in the borderline marking column

	Borderline	Clear Pass
Beginning the consultation	Begins by explaining the x-ray findings	Begins by establishing the reason for the consultation<br><br>Asks about the patient's current concerns
Why do I still have pain in my wrist?	Correctly identifies fracture on the x-ray and conveys this to the patient	Starts by asking the patient what they know so far<br><br>Explains that there is a fracture of the scaphoid<br><br>Explains that the bone is still in the correct position (undisplaced)
Why was I previously incorrectly told there was no	Apologises for the miscommunication<br><br>Explains that the fracture	Explains that this is the reason the patient was asked to come back to the clinic

fracture	sometimes is not visible on early x-rays	
What happens now? Will I need surgery?	Explains that a cast will be needed	Explains that as the bone is not displaced in position, non-surgical options are available initially
How soon can I remove the cast/splint?	Explains that the cast will be needed for around 6 weeks	Explains that a repeat x-ray will be taken to ensure healing<br><br>Explains that if the fracture is not treated for the correct length of time, there is a risk the bone may die (avascular necrosis of the scaphoid)
Conclusion	Concludes the consultation	Summarises the discussion and further follow up<br><br>Asks if there are any more questions
