# Supplementary material for: Is online objective structured clinical examination teaching an acceptable replacement in post-COVID-19 medical education in the United Kingdom?: a descriptive study
Source: J Educ Eval Health Prof. 2022 Nov 7;19:30. doi: 10.3352/jeehp.2022.19.30 (PMC9807458; doi:10.3352/jeehp.2022.19.30)
Supplement: Supplementary file 6 — Supplement 5. Surgical OSCE-Focused Teaching Station 4: 10 minutes—Stomas and Fluids for 10 minutes, executed by the University College London (UCL) Surgical Society with students from UCL Medical School, between February and May 2021. [file jeehp-19-30-suppl5.pdf]

Station 4: 10 minutes

Stomas and Fluids

Learning Objectives:

- Identify and understand the features differentiating types of stoma
- To understand the basics of fluid prescribing and be able to adapt these to a specific patient's requirements

Task 1: Stomas

- Spend 3 minutes reading the two patient descriptions and discussing with your tutor the characteristics of the stomas these patients are likely to have
    - *Most likely* site on abdomen
        - *Assuming average body habitus and no patient mobility issues*
    - Spouted or flushed
    - Loop or End
    - Type of output

Task 2: Post-operative fluid prescribing

- Spend 5 minutes calculating and discussing the prescribing task
- Use the information on the task to help you calculate this

Created by: Aniket Bharadwaj, Vashist Motkur and Nimalesh Yogarajah

Task 1: Stomas

Patient 1

A 49 year old man presents to A&E with fever and abdominal pain and severe diarrhoea. A previous colonoscopy showed submucosal lesions from the rectum up to the caecum. On this admission, an abdominal x-ray showed 7cm dilatation of peripherally located bowel loops. Due to the extent of previous disease, colorectal surgeons advised a panproctocolectomy.

Patient 2

A 23 year old woman with known ulcerative colitis presented to A&E with new onset bloody diarrhoea and severe abdominal pain, lasting 4 days. Investigation with colonoscopy and CT colonogram revealed colitis in the middle third of the distal colon. Colorectal surgeons proceeded to remove the affected portion of bowel and do a primary anastomosis. A temporary stoma is formed more proximally to protect the anastomosis.

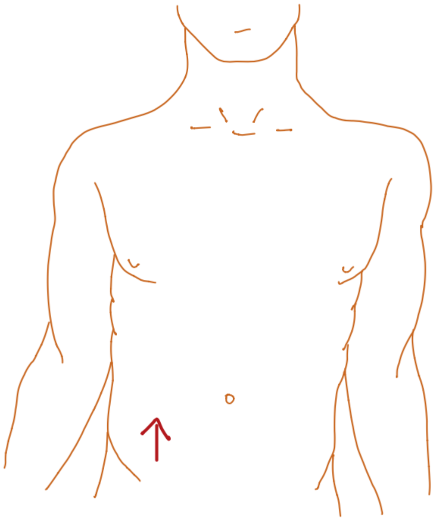

RIF

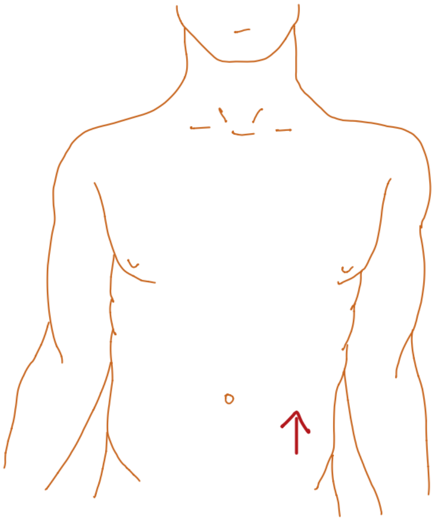

LIF

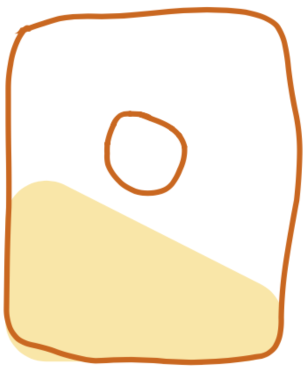

Liquid

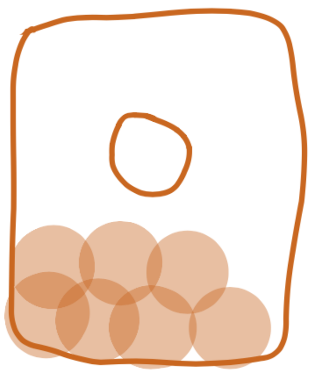

Solid

DISCUSS YOUR ANSWERS with the tutor:

	Site	Spouted?	Loop?	Type of output
Patient 1				
Patient 2				

Created by: Aniket Bharadwaj, Vashist Motkur and Nimalesh Yogarajah

Answers

Key learning point: identify different features of ileostomies vs colostomies

Patient 1:

Ileostomy (complete removal of the colon)

Site - right iliac fossa

Spouted - The ileum is everted on itself to form a spout since it contains activated digestive enzymes and discharges almost continuously which can excoriate and digest the skin. The stoma bag should be elevated 2-3 cm from the skin to ensure the effluent passes directly into a stoma bag with minimal contact with skin

Loop - End

Output - Serous liquid.

Patient 2:

Colostomy

Site - left iliac fossa

Spout - sutured flush to the skin, with a slight pouch to prevent retraction after weight gain. Not directly corrosive to the skin

Loop - Loop Colostomy (double barren lumen seen)

Output - Solid output that is discharged intermittently.

Diagrams of ileostomy and colostomy appearances redacted as permission not obtained for publication

Task 2: Post-operative fluid prescribing

Student Brief

Patient 2 is Barry McLeod, a 70 year old gentleman who recently underwent a right hemicolectomy with defunctioning colostomy, for colonic cancer.

He weighs 90kg and has been appropriately resuscitated. His serum electrolytes are in the normal range.

Please use the fluid balance chart below to calculate how much fluid he needs replaced, combined with his maintenance requirements as he remains nil by mouth after his hemicolectomy.

Discuss with your group what you would prescribe as the appropriate fluids for the next 24 hours.

<u>Fluid Balance Chart for the past 24 hours</u>

**Time**	**Input**	**Output**	**Time**	**Input**	**Output**
19:00	Fluid - 1000ml	Urine - 300ml	07:00		
20:00		Vomit - 200ml	08:00		Stoma loss - 400ml
21:00			09:00		Urine (catheter) - 400ml
22:00		Vomit - 300 ml	10:00	Fluid resus - 500ml	
23:00		Stoma loss - 400ml	11:00		
00:00			12:00		Stoma loss - 300ml
01:00			13:00	Fluid resus - 500ml	
02:00			14:00		Urine (catheter) - 300ml
03:00			15:00		
04:00		Urine - 300ml	16:00		Stoma loss - 300ml
05:00			17:00		
06:00	Fluid - 1000ml		18:00		Urine (catheter) - 200ml
**TOTAL**	<u>2000ml</u>	<u>1500ml</u>	**TOTAL**	<u>1000ml</u>	<u>1900ml</u>

<u>Empty chart</u>

Created by: Aniket Bharadwaj, Vashist Motkur and Nimalesh Yogarajah

Infusions

	Fluid				Drug added (If any)		Signature	Bleep	Given
Date	Type	Volume	Duration	Route	Name	Dose			
A	B	C	D	E	F	G			

Answers

Total fluid input = 3000ml
Total fluid output = - 3400ml
Net fluid balance = - 400ml

Daily requirements for a 90kg man:

- Water: **30ml/kg/day x 90kg = 2700ml/day**
    - In practice, the closest multiple of 500ml is given to most patients

- Sodium/potassium/chloride: **1mmol/kg/day x 90kg = 90mmol/day**
    - The maximum concentration of potassium that can be given is 40 mmol/L
    - Potassium comes in ready made bags of 40 mmol or 20 mmol
        - This is the case for 0.9% NaCl, Dextrose and Dextrose-saline
        - Hartmann's is a pre-made composition of Na^+, K^+, Cl^-, Bicarb, Ca^{2+} - therefore you cannot add extra K^+ to Hartmann's and as such it should only be used for resuscitation, not maintenance
        - For practical purposes, the closest multiple of 20mmol is usually given for most patients

- Glucose: **50-100g/day**

Given a net loss of 400ml, the patient would require 3100ml in total. As mentioned above, in practice we would give 3L.

	Bag 1	Bag 2	Bag 3	Notes
A	Date	Date	Date	
B	5% Dextrose	5% Dextrose	0.9% Sodium Chloride	These bags can be prescribed in any order
C	1L	1L	1L	
D	8 hours	8 hours	8 hours	
E	IV	IV	IV	
F	Potassium Chloride	Potassium Chloride	Potassium Chloride	
G	40 mmol	20 mmol	20 mmol	These can be added in any order, as long as they are 40mmol or 20mmol and do not exceed 90mmol

Mark Scheme

Fail: When a student does not meet majority of the points in the borderline marking column

Borderline	Clear Pass
Calculates net fluid balance by subtracting output from input.<br><br>Is able to correctly fill out an IV infusion section of the drug chart, including fluid type, volume, route and duration<br><br>Prescribes at least one appropriate bag of fluid (e.g. 0.9% NaCl, 5% dextrose)	+ Takes into account daily requirements of various solutes including chloride, potassium and glucose and prescribes dextrose bags and potassium additions accordingly<br><br>+ Incorporates net fluid balance and maintenance fluids when prescribing fluids for the patient, correctly calculating how much fluid the patient must have over 24 hours

**Solution**	**Na^+ (mmol/L)**	**Cl^- (mmol/L)**	**K^+ (mmol/L)**	**HCO_3^- (mmol/L)**	**Ca^{2+} (mmol/L)**	**Glucose (mmol/L)**	**Osm (mmol/L)**
Plasma	135-145	95-105	3.5-5.3	24-32	2.2-2.6	3.5-5.5	275-295
0.9% NaCl	154	154	0 or 20 or 40	0	0	0	308
5% Glucose	0	0	0 or 20 or 40	0	0	278 (50g)	278
0.18% NaCl / 4% Glucose	31	31	0 or 20 or 40	0	0	222 (40g)	284
Hartmann's	131	111	5	29	2	0	278

Adapted from NICE Guidance CG174

Hartmann's is of similar composition to Plasma, hence it is commonly used for resuscitation and maintenance - however the issue with it is that you cannot add extra K^+. For this reason you have to be careful about using only Hartmann's for maintenance, as the patient may not receive enough K^+ (and glucose).

Giving a combination of NaCl and Glucose (either as separate bags or as the combined 0.18% NaCl / 4% Glucose) and choosing the appropriate K^+ additive will allow you to best meet the patient's daily maintenance requirements.
