# Supplementary material for: Is online objective structured clinical examination teaching an acceptable replacement in post-COVID-19 medical education in the United Kingdom?: a descriptive study
Source: J Educ Eval Health Prof. 2022 Nov 7;19:30. doi: 10.3352/jeehp.2022.19.30 (PMC9807458; doi:10.3352/jeehp.2022.19.30)
Supplement: Supplementary file 7 — Supplement 6. Surgical OSCE-Focused Teaching Station 5: 5 minutes—Red Hot Joint for 5 minutes, executed by the University College London (UCL) Surgical Society with students from UCL Medical School, between February and May 2021. [file jeehp-19-30-suppl6.pdf]

Station 5: 5 Minutes

Red hot joint

Learning Objectives:

- Learn how to take a focused history
- Practise documenting management plans
- Have an understanding of how to manage a red hot swollen joint

Task 1: Patient consultation

- Spend 5 minutes taking a focussed history and vocalising your management plan to the group
  - One student to take the history and explain the management
  - One student acts as the patient
  - One student is the examiner

Task 1: Patient consultation

Student Brief

You are a 4th Year Medical Student on placement in the A&E Minors department.

John Stone is a 42 year old man who has come in complaining of pain in his right knee.

1. Please take a focused history from this patient
2. At the end explain your plan to the examiners (as if you were documenting it in the patient notes)

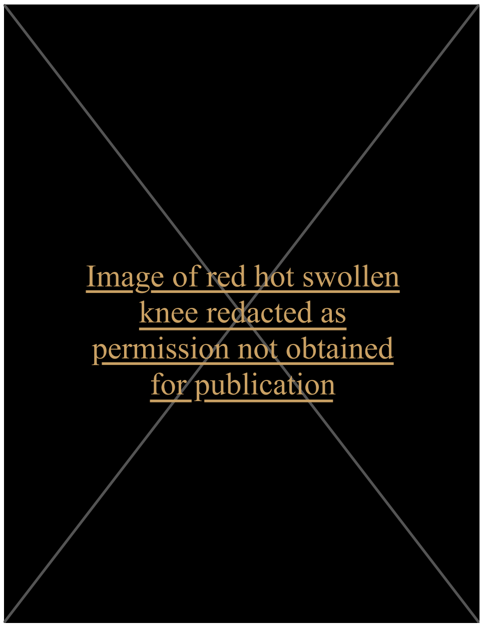

Patient Brief

You are a 42 year old male named John Stone who has come in due to a red hot swollen **right** knee joint.

HPC:
- The pain started 2 days ago
- Right knee has been swollen and painful.
- This has been worsening - and now I can't walk properly.
- No injury or trauma to the joint
- No other joints are painful
- Pain is 8/10 atm but that's because I've had paracetamol
- If probed about temperature/fever - say nurse measured it at 38.6

Travelling: Flew to philippines on a work visit to a steel factory
Sexual: mention that you are married for the past 18 years

Past medical history:
- Well controlled diabetes
- Hypertension

Past surgical history:
- Left knee replacement 5 years ago
- Appendicectomy when you were a teenager

Drug history: Metformin, ramipril

Allergies: Codeine

Family history: Nil

Social history: Smokes 5 day, 2 bottles of beer over the weekend. Steel factory manager. Lives in a house with wife and son.

Examiner Brief

Fail: When a student does not meet majority of the points in the borderline marking column

Borderline	Clear Pass
Mentions parts of "DWARFS" and socrates<br>Asks about trauma<br>Asks about PMH<br><br>Drug Hx + allergies asked<br>Smoking and alcohol asked	+ Good dwarfs and socrates<br>+ Eludes to immunosuppression as a risk factor<br>+ Asks about past surgical history<br>+ IVDU<br>+ Traveling history<br>+ Sexual history
Plan:<br>• Bloods including cultures<br>• Joint aspiration<br>• Antibiotic Tx<br>• X-ray of joint<br>• Pain relief<br>• Refers to senior	Plan:<br>• Bloods: including FBC, CRP, ESR, cultures<br>• Aspirate and send to MC&S<br>• Start broad spectrum antibiotics as per local guidelines<br>○ Checking patient's allergy status<br>• X-ray of the right and left knee to compare if no previous films<br>• Appropriate pain relief<br>• Refer to ortho

Candidate essentially needs to recognise:

This is a purulent intra-articular infection, brought from another area via the blood (haematogenous seeding). If it is not treated, it can lead to destruction of the joint.

A differential could be osteomyelitis, however, given that it's a joint that is affected rather than long bones/pelvis/spine - it is less likely to be the case.
