# Supplementary material for: Is online objective structured clinical examination teaching an acceptable replacement in post-COVID-19 medical education in the United Kingdom?: a descriptive study
Source: J Educ Eval Health Prof. 2022 Nov 7;19:30. doi: 10.3352/jeehp.2022.19.30 (PMC9807458; doi:10.3352/jeehp.2022.19.30)
Supplement: Supplementary file 8 — Supplement 7. Surgical OSCE-Focused Teaching Station 6: 10 minutes—DR ABCDE for 10 minutes, executed by the University College London (UCL) Surgical Society with students from UCL Medical School, between February and May 2021. [file jeehp-19-30-suppl7.pdf]

Station 6: 10 minutes

DR ABCDE

Learning Objectives:

- Learn how to assess an acutely unwell patient using a DR ABCDE approach

Task 1: DR ABCDE

- You can take up to 10 minutes to assess the patient. Talk through what steps you would take.
- One person will be the candidate, one person will be the patient, one person will be the nurse/examiner

Task 1: Assessment of the acutely unwell patient

Student brief

You are a 4th year medical student on the surgical ward. A nurse calls you over to the bed of Barry White, a 65 year old gentleman who looks unwell.

Please help her assess the patient.

Nurse brief

You are a nurse on the ward. You found Barry White deteriorating for the last 20 minutes, and he is very unwell and in distress. You know that he is 2 days post-op for a right hemicolectomy. Past medical history includes T2DM, hypertension, and colorectal carcinoma of the descending colon. If asked, you have not done any basic observations yet.

Please refer to the scenario below as the candidate progresses through the assessment.

Patient brief

You are an unwell and distressed patient on the ward who had a right hemi-colectomy 2 days ago.

Please refer to the scenario below as the candidate progresses through the assessment.

Scenario

	**Initial assessment**	**On re-assessment** *(only mention obs changes if student asks for them)*
**D, R, obs**	No danger<br>Responds in full sentences when spoken to: doesn't feel well	No change
**A**	No swelling or airway obstruction<br>Able to talk in full sentences	No change
**B**	There is some difficulty in breathing - you are breathing quite fast and having to use accessory muscles<br><br>Equal chest expansion<br><br>No wheeze or crackles<br><br>RR 24, 94% on air	On 15L/min:<br>O2 sats improve to 98%, RR 20

Created by: Aniket Bharadwaj, Vashist Motkur and Nimalesh Yogarajah

**C**	Pale and clammy<br>Cool peripheries<br><br>Peripheral pulse is thready, central pulse is normal volume, regular<br><br>Cap refill: 4 seconds<br>HS 1+2+0<br>BP: 89/60<br>Pulse: 125<br><br>Temperature: 38.5C<br><br>ECG shows sinus tachycardia<br><br>Lactate on VBG 2.9<br><br>There is a catheter in situ and has drained 100ml in the past hour	After STAT IV fluids:<br><br>BP 112/65<br>HR 108<br><br>Warmer peripheries, stronger peripheral pulse with cap refill now 3 seconds
**D**	Alert and pupils are equal and reactive<br><br>GCS 15/15<br>BM 5.0	No change
**E**	Abdomen is soft and not tender.<br>Patient is pale but no jaundice<br><br>Calves are soft and not tender<br><br>No rashes or obvious external bleeding<br><br>No facial droop, moving all four limbs spontaneously. Normal sensation in upper and lower limbs bilaterally.	No change

	**Points to consider (not specific to this scenario)**
**D, R, obs**	Danger, Response<br>Ask for initial basic obs<br>Call for appropriate help
**A**	<u>Look</u>: swollen lips/ tongue, obvious obstruction (e.g. vomit)<br><br><u>Feel</u>: on cheek for presence of air<br><br><u>Listen</u>: stridor, wheeze, gurgling, snoring. Talking?<br><br><u>Treat</u>: aspiration, physical removal of obstruction (must always be able to visualise end of tool), airway adjuncts.
**B**	<u>Look</u>: sweating, cyanosis, pursed lips, nasal flaring. Use of accessory muscles, symmetrical, tracheal tug. Ankle/ sacral oedema<br><br><u>Feel</u>: clamminess, tracheal deviation, expansion<br><br><u>Listen</u>: percussion, breath sounds<br><br><u>Measure</u>: **SOCRAP** (sputum, O2 sats, CXR, RR, ABG, PEF).<br><br><u>Treat</u>: High flow O2 (15L/min through non-rebreathe mask), SABA/SAMA nebulisers
**C**	<u>Look</u>: pallor, peripheral cyanosis, clamminess, JVP, pedal oedema, DVT, skin turgor/mucous membranes<br><br><u>Feel</u>: hands, temperature, clamminess, pulse – regular?<br><br><u>Listen</u>: heart sounds, lung bases<br><br><u>Measure</u>: **BUTCHE** (BP, urine output [*and dipstick*], temperature, capillary refill, heart rate, ECG<br><br><u>Treat</u>: Two wide-bore cannulae:<br>1. Routine bloods, blood cultures, VBG for lactate (or ABG if indicated), group and save + crossmatch;<br>2. If hypovolemic/low BP → **<u>fluids</u>** (e.g. 500ml Hartmann's in 15 mins).<br>3. Catherise<br>4. Broad spectrum antibiotics if indicated

**D**	AVPU/GCS<br>Capillary blood glucose.<br>Pupils are equal and reactive to light
**E**	*Top to toe examination for rashes, wounds, bleeds, DVT etc.*<br><br>***This may drive you to actually carry out a specific examination, e.g. focussed abdominal or neurological examination.***<br><br>Probe student if needed - what would you do once you've done your initial management<br><u>After initial management</u>:<br>1. Reassess!!<br>2. Document in notes<br>3. SBAR handover to senior<br>4. State you would monitor observations regularly

Mark Scheme

Fail: When a student does not meet majority of the points in the borderline marking column

Borderline	Clear Pass
Able to assess the patient by conducting examinations of the relevant body systems<br><br>As a minimum, passing candidates should be able to assess for at least three clinical signs in each of the A/B/C/DE sections of the assessment<br><br>Able to start treating the patient based on derangement of observations and clinical signs, including:<br>• Administering appropriate oxygen<br>• Fluid resuscitation<br>• Considering antibiotics due to pyrexia	+ Organising the assessment with structured approach, such as DRABCDE with a 'look, feel, measure, treat' structure<br><br>+ Successfully identifies patient likely to be septic, and mentions initiation of the **sepsis 6**:<br>• Catheterise patient + urine output monitoring<br>• Blood gas to measure lactate<br>• Obtains blood cultures for sensitivities<br>• Administers oxygen<br>• Gives fluids<br>• Starts antibiotics

Candidates will recognise the need to escalate to a senior colleague for advice on management, and to document the assessment in the patient's notes	+ Candidates with a clear pass will also reassess observations to evaluate if their treatments have proven to be effective<br><br>+ Initial call for help is appropriate. If the candidate has not assessed the patient yet, then calling for a nurse to help with the assessment will be appropriate. If an airway issue is encountered, then the anaesthetist should be called. If the patient is peri-arrest then the PERT/medical emergency team should be called. In a cardiac arrest the crash team should be called. In most other situations, once the candidate has done all that they can, a senior should be called.
